# Transcriptional profiling of *Actinobacillus pleuropneumoniae *during the acute phase of a natural infection in pigs

**DOI:** 10.1186/1471-2164-11-98

**Published:** 2010-02-08

**Authors:** Vincent Deslandes, Martine Denicourt, Christiane Girard, Josée Harel, John HE Nash, Mario Jacques

**Affiliations:** 1 Groupe de Recherche sur les Maladies Infectieuses du Porc, Faculté de médecine vétérinaire, Université de Montréal, St-Hyacinthe, Canada; 2 Centre de Recherche en Infectiologie Porcine, Faculté de médecine vétérinaire, Université de Montréal, St-Hyacinthe, Canada; 3 Département de sciences cliniques, Faculté de médecine vétérinaire, Université de Montréal, St-Hyacinthe, Canada; 4 Département de pathologie et microbiologie, Faculté de médecine vétérinaire, Université de Montréal, St-Hyacinthe, Canada; 5 Institute for Biological Sciences, National Research Council of Canada, Ottawa, Canada; 6 Current address: Office of Biotechnology, Genomics and Population Health, Public Health Agency of Canada, Ottawa, Canada

## Abstract

**Background:**

*Actinobacillus pleuropneumoniae *is the etiological agent of porcine pleuropneumonia, a respiratory disease which causes great economic losses worldwide. Many virulence factors are involved in the pathogenesis, namely capsular polysaccharides, RTX toxins, LPS and many iron acquisition systems. In order to identify genes that are expressed *in vivo *during a natural infection, we undertook transcript profiling experiments with an *A. pleuropneumoniae *DNA microarray, after recovery of bacterial mRNAs from serotype 5b-infected porcine lungs. AppChip2 contains 2033 PCR amplicons based on the genomic sequence of *App *serotype 5b strain L20, representing more than 95% of ORFs greater than 160 bp in length.

**Results:**

Transcriptional profiling of *A. pleuropneumoniae *recovered from the lung of a pig suffering from a natural infection or following growth of the bacterial isolate in BHI medium was performed. An RNA extraction protocol combining beadbeating and hot-acid-phenol was developed in order to maximize bacterial mRNA yields and quality following total RNA extraction from lung lesions. Nearly all *A. pleuropneumoniae *transcripts could be detected on our microarrays, and 150 genes were deemed differentially expressed *in vivo *during the acute phase of the infection. Our results indicate that, for example, gene *apxIVA *from an operon coding for RTX toxin ApxIV is highly up-regulated *in vivo*, and that two genes from the operon coding for type IV fimbriae (APL_0878 and APL_0879) were also up-regulated. These transcriptional profiling data, combined with previous comparative genomic hybridizations performed by our group, revealed that 66 out of the 72 up-regulated genes are conserved amongst all serotypes and that 3 of them code for products that are predicted outer membrane proteins (genes *irp *and *APL_0959*, predicted to code for a TonB-dependent receptor and a filamentous hemagglutinin/adhesin respectively) or lipoproteins (gene *APL_0920*). Only 4 of 72 up-regulated genes had previously been identified in controled experimental infections.

**Conclusions:**

These genes that we have identified as up-regulated in *vivo*, conserved across serotypes and coding for potential outer membrane proteins represent potential candidates for the development of a cross-protective vaccine against porcine pleuropneumonia.

## Background

*Actinobacillus pleuropneumoniae *is the causative agent of porcine pleuropneumonia, a highly contagious respiratory disease that causes great economic losses worldwide [1]. Transmission occurs through aerosol or close contact with infected animals or asymptomatic carriers, and can affect pigs of all ages [2]. In the acute phase, the disease is often lethal in 24 to 48 h, and leads to the formation of extensive fibrinohemorrhagic lung lesions. Animals that survive the infection often become healthy carriers, and develop localized necrotizing lesions associated with pleuritis [3]. Fifteen different serotypes can be identified based on differences in capsular polysaccharides. While serotypes 1 to 12 and 15 usually belong to biotype 1, which contains strains that are NAD-dependent, serotypes 13 and 14 are usually NAD-independent and belong to biotype 2 [4]. However, biotype 2 variants of serotypes 2, 4, 7 and 9 have been reported [5-7]. Despite years of research, mechanisms involved in *A. pleuropneumoniae *pathogenicity are still not fully understood. While many virulence factors have been identified, such as lipopolysaccharides, capsular polysaccharides, Apx toxins and iron acquisition systems [4], or suggested, like biofilm formation [8], autotransporter adhesin [9] and autotransporter protease synthesis [10], very little is known about the overall contribution of each component to the infection process.

In order to gain new insight into the disease, researchers have relied on techniques that allow for the identification of genes expressed in bacteria during infection of the host [11], namely *In Vivo *Expression Technology (IVET), Signature Tagged Mutagenesis (STM) and Selective Capture of Transcribed Sequences (SCOTS). First, an IVET experiment with *A. pleuropneumoniae *led to the identification of 10 loci (termed *ivi*) transcribed *in vivo *[12] and highlighted the importance of genes involved in branched-chain amino acid (BCAA) synthesis [13,14]. Later, over the course of two STM experiments conducted with *A. pleuropneumoniae*, nearly 3000 mutants were screened *in vivo *[15,16]. Both experiments showed that iron acquisition was highly important for the virulence of *A. pleuropneumoniae*, with the identification of mutants impaired for the ExbB-ExbD-TonB energy-transducing system. The study by Sheehan et al [16], lead to the identification of a second set of *exbB-exbD-tonB *genes, and it was determined that inactivation of *tonB2 *and not *tonB1 *lead to attenuation *in vivo *[17]. More recently, SCOTS experiments were conducted with *A. pleuropneumoniae*, at two different stages of infection: at the end of the acute stage, in necrotic lung tissues (7 days post-infection) [18], and in the chronic stage (21 days post-infection) [10]. These studies demonstrated the importance of anaerobic metabolism and HlyX-regulated mechanisms during *A. pleuropneumoniae *infection in the lungs, and led to the identification and characterisation of a maturation autotransporter protease, AasP, responsible for cleavage and release of fragments of OmlA from the cell surface [19]. The HlyX regulon was characterized recently, and iron-regulated protein B (FrpB) was identified as a potentially important virulence factor up-regulated when *A. pleuropneumoniae *is grown in oxygen-deprived environments [20]. Although these *in vivo *studies have been very valuable in gaining a better understanding of the mechanisms involved in *A. pleuropneumoniae *pathogenesis, limitations associated with the techniques used lead only to a partial overview of the transcriptional events taking place *in vivo*.

DNA microarrays have been used to determine the complete transcriptomic profile of microorganisms *in vitro*, but technical drawbacks that are associated with the use of this technique *in vivo*, such as the qualitative and quantitative recovery of bacterial RNA from host tissues, explain why the use of this technique to monitor bacterial infections has been limited [21]. While various cell culture, *ex-vivo *or mouse infection models have been used, *in vivo *experiments can provide more accurate information on bacterial adaptation and virulence when the natural host is studied. To the best of our knowledge, only two genome-wide transcriptional profiling experiments using DNA microarrays have been conducted with bacterial pathogens in their natural host, during a natural infection. LaRocque et al. have evaluated the transcriptome of *Vibrio cholerae *in the early and late stages of the disease by collecting stools of naturally infected patients [22]. Son et al. used fresh sputum samples from a patient with cystic fibrosis to study the *in vivo *transcriptome of *Pseudomonas aeruginosa *[23]. Additionally, Agarwal et al. used a custom-made Fur and iron regulon microarray to investigate the expression of these genes during gonococcal infection in women [24].

In this study, we have conducted *in vivo *transcript profiling with DNA microarrays of *A. pleuropneumoniae *during the acute phase of a natural infection in pigs. Our results indicate that 150 genes are differentially expressed during the acute phase of infection in the host. Of these, 72 were up-regulated, and three of these encode lipoproteins or OMPs that are well-conserved among strains of *A. pleuropneumoniae*.

## Results and discussion

### Animal samples

While working with samples from commercial herds means that we are working with disease cases that are representative of what is really happening in the field, it also means that limited amounts of material is available. During the course of our experiments, we were able to collect samples from three different animals from the same herd, before antibiotherapy was undertaken to eradicate the infection. All the animals that were sampled had presented symptoms of the disease for less than 24 h. Two animals died as a result of the infection, and were sampled a few hours after death. Those samples were not used for the microarray experiments since significant transcriptomic changes might have occurred between the time of death and the time of sampling. However, preliminary microscopic observations were made and our RNA extraction protocol was tested with those samples. The last animal was euthanized on site by a veterinarian, and sampled less than 5 minutes after death. The infectious field strain was isolated from this animal. While it can be argued that different animals might react differently to an infection by *A. pleuropneumoniae*, and therefore elicit different transcriptional changes in the pathogen, we are confident that the results that were obtained reflect accurately the conditions encountered by *A. pleuropneumoniae *in its natural host following acute infection.

### Macroscopic and microscopic observations

**Figure 1:**
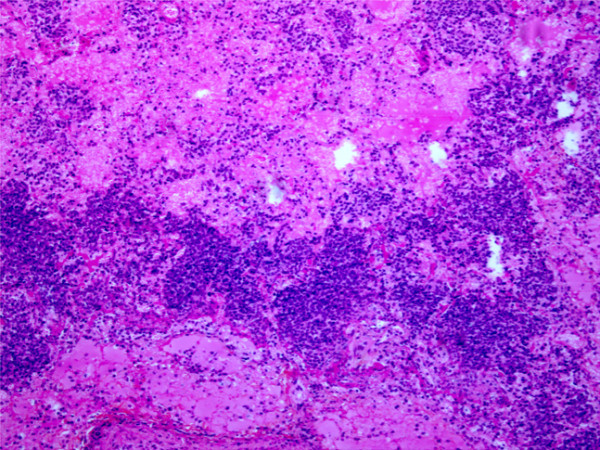
**HPS staining of paraffin-embedded infected lung tissue sections, at 100× magnification**. Severe fibrinohemorragic pneumonia with infiltration of oat cells.

Clinical signs and macroscopic and microscopic examinations were consistent with acute porcine pleuropneumonia. Clinical signs ranged from depression to respiratory distress, while macroscopic observation of deceased pigs revealed lobar fibrinohemorrhagic pleuropneumonia, along with necrotic lesions and the accumulation of bloody fluid in the thorax. On microscopic examination, multiple pulmonary necrotic areas surrounded by or containing oat cells were present, as well as fibrin accumulation in the alveolar lumen, interlobular septa and pleura (Figure 1). The infectious field strain that was isolated from the lung tissues was named 896-07, and serotyping analysis showed that it belongs to serotype 5b, one of the most prevalent in North America [4].

### Comparative genomic hybridizations

CGH experiments conducted previously in our laboratory have shown that the majority of genes in the reference 5b L20 strain were conserved in reference strains and fresh field isolates. Conversely, 205 genes were identified as divergent in at least one strain, including 39 that are either phage or transposon related out of the 2033 ORFs represented on the microarray, which represents 10.1% of divergent genes [25]. As anticipated, clusters of genes associated with LPS/capsule biosynthesis and toxin production showed variations.

**Figure 2:**
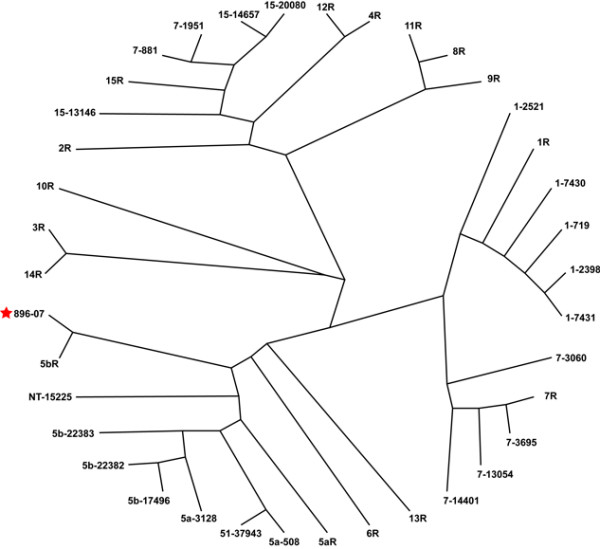
**Hierarchical clustering of reference and field strains of *A. pleuropneumoniae*, including strain 896-07, based on CGH data obtained with AppChip2**. Strains are identified according to "serotype-strain number". Field strains are the same as those used by Gouré et al[25]. R: reference strain. NT: Non-typeable.

AppChip2 was designed using the complete genome sequence from the serotype 5b reference strain L20. In order to validate its use to assess transcriptomic events taking place with our infectious field strain during the infection, we first used it to analyze the gene content of the 896-07 field strain . The CGH data obtained with strain 896-07 was added to that obtained with other field and reference strains [25] in order to generate a strain dendogram (Figure 2). Serotyping data correlates well with genetic data since strain 896-07 forms a cluster with the serotype 5b reference strain L20. The same clusters as those observed previously could be found in our dendrogram [25].

Only 28 genes were identified as divergent/absent in our field strain, when compared to the reference serotype 5b L20 strain (Table 1). This represents only 1.43% of all the genes (1954) that are represented on AppChip2 [25]. Out of these 28 genes, 20 can be found in two major regions of the genome: 14 putative ORFs code for phage structures, while 6 are located in the *A. pleuropneumoniae *tight adherence (*tad*) locus. In *Aggregatibacter actinomycetemcomitans*, a close relative of *A. pleuropneumoniae*, the *tad *locus is involved in adherence to biological and abiological surfaces [26]. In *A. pleuropneumoniae*, genes from this locus were shown to be up-regulated during adhesion to lung epithelial cells *in vitro *[9]. The other important locus for gene divergence/absence was composed of phage-related features, and these were shown to be divergent during inter and intra-serotype comparisons in previous experiments [25]. Some genes might also be present in the genome of strain 896-07 and absent in reference strain L20, and therefore absent on AppChip2.

**Table 1:** Genes that are divergent or absent in the *A. pleuropneumoniae *896-07 field strain after comparison with the genome of serotype 5b reference strain L20

R	Locus Tag	Gene	Description	Log_2 _ratio
P	APL_0495	*APL_0495*	Putative DNA-methyltransferase	-1.135

P	APL_0496	*APL_0496*	Hypothetical protein	-2.324

P	APL_0499	*APL_0499*	Hypothetical protein	-1.166

P	APL_0500	*APL_0500*	Hypothetical protein	-1.071

P	APL_0501	*APL_0501*	Possible DNA methylase	-1.418

P	APL_0502	*APL_0502*	Hypothetical protein	-1.794

P	APL_0503	*rusA*	Predicted Endodeoxyribonuclease RusA	-1.065

P	APL_0505	*APL_0505*	Putative endolysin	-1.306

P	APL_0509	*APL_0509*	Bacteriophage capsid protein	-1.825

P	APL_0518	*APL_0518*	Hypothetical protein	-3.227

P	APL_0520	*APL_0520*	Hypothetical protein	-1.101

P	APL_0522	*APL_0522*	Phage-related tail assembly protein K	-1.635

P	APL_0523	*APL_0523*	Putative phage tail assembly protein	-1.533

P	APL_0524	*APL_0524*	Predicted phage tail protein	-1.097

T	APL_0547	*tadF*	Tight adherence protein F	-0.909

T	APL_0550	*tadC*	Tight adherence protein C	-1.528

T	APL_0551	*tadB*	Tight adherence protein B	-1.917

T	APL_0552	*tadA*	Tight adherence protein A	-1.781

T	APL_0553	*tadZ*	Flp operon protein D	-1.991

T	APL_0556	*rcpC*	Flp operon protein C	-1.815

O	APL_0427	*adhA*	NADP-specific glutamate dehydrogenase	1.081

O	APL_0445	*ykgF*	Putative electron transport protein	-1.144

O	APL_0560	*rhlB*	ATP-dependent RNA helicase RhlB	-2.063

O	APL_0672	*APL_0672*	Hypothetical protein	0.927

O	APL_1959	*adhI*	Alcohol dehydrogenase 1	1.015

O	APL_2001	*APL_2001*	Hypothetical protein	-1.618

O	APL_2011	*aldA*	Putative aldehyde dehydrogenase AldA	1.813

O	APL_2038	*lolC*	Lipoprotein-releasing system transmembrane protein LolC	-0.915

Genes are ordered according to their location in the *A. pleuropneumoniae *genome (locus tag). Two important genomic regions (R) were found in this list: a phage region (P), and the *tad *locus (T).

R: genomic region. P: phage region. T: *tad *locus. O: other region

### RNA extraction

Tissue samples were taken where lung lesions were present to ensure that bacterial yields would be optimal. A beadbeating step was performed prior to a standard hot acid-phenol-chloroform RNA extraction protocol to extract bacterial RNA from infected lung tissues. Tissues were homogenized with 1.0 mm zirconia/silica beads to release but not lyse bacterial cells from the tissue. This size of beads is too large to lyse bacterial cells (Biospec, personal communication). Following homogenization, serial dilutions of the resulting supernatant were plated in order to determine an approximate number of CFU per gram of tissue. Our results indicated that *A. pleuropneumoniae *could easily be isolated in pure culture from lung tissues of all animals, and that the number of CFU varied from 1 × 10^6 ^to 1 × 10^7 ^per gram of infected tissue. Centrifugation of homogenized samples enabled the removal of most cellular debris and the recovery of bacteria. Approximately 20 to 45 μg of total RNA could routinely be extracted from approximately 2 g of necrotic lung tissue, with no apparent contamination by eukaryotic RNA, as observed on agarose gel when using bacterial RNA extracted from BHI broth and eukaryotic RNA recovered from porcine lung epithelial cells (SJPL cell line) [27] as on-gel controls (data not shown). Nevertheless, the MicrobENRICH kit was still used to remove any possible trace of remaining eukaryotic RNA, and treatments with TurboDNase removed traces of contaminating DNA. No trace of bacterial or host DNA could be detected following PCR amplification with primers for gene *ompW *and for pig mitochondrial DNA. We propose that this combination of techniques could prove useful in isolating pathogens from other tissues as well.

### In vivo transcript profiling

Before investigating the *in vivo *transcript profile of *A. pleuropneumoniae*, we used self-self hybridizations in order to quantifiy the dye bias effect after hybridization on AppChip2. Duplicate experiments showed a R^2 ^correlation factor of 0.996 between the intensity detected for the Cy3 labelled and Cy5 labelled probes, and only two genes seemed to qualify as obvious outliers (data not shown). Both genes, APL_1141 and APL_0484, were not identified as differentially expressed during the course of a natural infection.

While designing the microarray experiments, growth in BHI medium to an optical density of 0.3 at a 600 nm wavelenght was chosen as our reference condition. This condition was not only chosen for practical reasons, but also to fulfill two precise objectives: first, to allow for the identification of genes that, while important to the infection process, have not been identified previously *in vitro *in rich medium, and second, to allow comparison of the results obtained *in vivo *to those obtained in our laboratory in other growth conditions by keeping the same reference [28-30]. Working with a limited amount of biological material meant that only one time-point in the infection could be investigated. In the acute stage of porcine pleuropneumonia, bacteria are likely in the exponential phase of growth. This is also the case in the control condition. The transcriptome of *A. pleuropneumonia *is certainly very different when the infection is in the chronic stage. Therefore, while our results are an accurate representation of the transcriptomic events that occur *in vivo *during the acute phase of the infection process as compared to those occuring *in vitro *in a rich culture medium, events occuring earlier of later in the infection process cannot be inferred.

**Figure 3:**
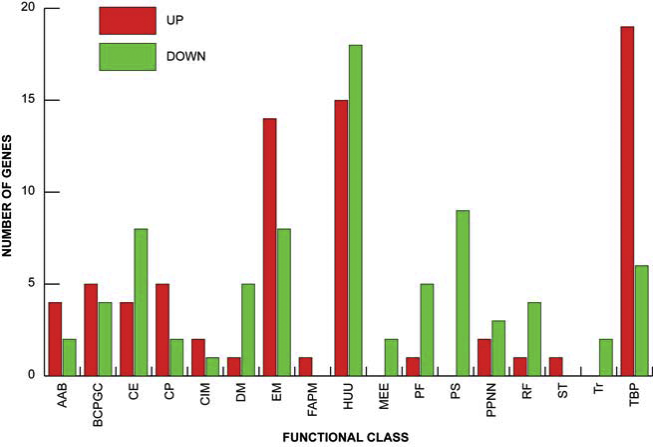
**Functional classification of *A. pleuropneumoniae *genes that are differentially expressed in infected pig lungs**. AAB, amino acid biosynthesis. BCPGC, biosynthesis of cofactors, prosthetic groups and carriers. CE, cell enveloppe. CP, cellular process. CIM, central intermediary metabolism. DM, DNA metabolism. EM, energy metabolism. FAPM, fatty acid and phospholipid metabolism. HUU, hypothetical/unclassified/unknown. MEE, mobile extrachromosomal element functions. PF, protein fate. PS, protein synthesis. PPNN, purines, pyrimidins, nucleosides and nucleotides. RF, regulatory functions. ST, signal transduction. Tr, transcription. TBP, transport and binding proteins.

*In vivo *transcript profiling experiments were performed on samples from three different lesion sites on the euthanized pig. Since this pig was euthanized on site by a veterinarian, the time laps between the death of the animal and tissue sampling was kept to a minimum. Following microarray hybridization and analysis, 86.7% of all coding sequences included on AppChip2 could be detected during our experiments, and 150 genes were significantly differentially expressed *in vivo *when their level of expression was compared to that seen *in vitro*, with a FDR of 4.25% over the course of three hybridizations: 72 of these were up-regulated, while 78 were down-regulated. Functional classification of these genes revealed that up-regulated genes mostly belonged to the "Transport and Binding Proteins" and "Energy Metabolism" classes, with respectively 17 and 14 genes (see Additional file 1 and Figure 3). Repressed genes mostly belonged to the "Protein Synthesis", "Energy Metabolism" and "Cell Envelope" functional classes. A large number of genes encoding "Hypothetical/Unknown/Unclassified" products were differentially expressed. Only ten out of the 150 differentially expressed genes (8.2%) could be found in the list of divergent or highly divergent/absent genes in at least one reference strain of *A. pleuropneumoniae*, as established by Gouré et al. [25]. Three of these are involved in surface structures, namely LPS and capsule, biosynthesis (*genes kpsF*, *cps5b *and *cpxC*), and two others are found in phage-associated regions (APL_0512 and APL_0524). Of the last five genes, four are divergent in only one strain (*hktE*, *fur*, *phoR *and *hisH*, respectively in reference strains from serotypes 8, 2, 15 and 7), and the APL_0999 gene codes for a hypothetical protein that is divergent in serotype 2, 7-11 and 14 reference strains.

#### a) Validation of microarray results by qRT-PCR

**Figure 4:**
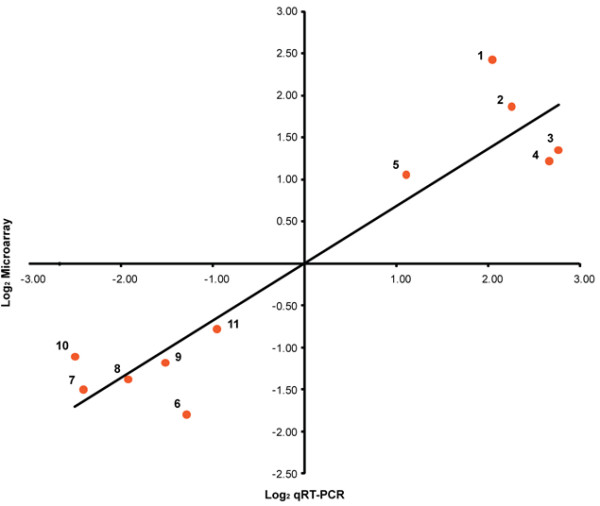
**Validation of microarray results by qRT-PCR**. Results from five up-regulated and six down-regulated genes are presented. Mean log_2 _ratios obtained during qRT-PCR experiments are plotted against the mean log_2 _ratios obtained with the microarrays. Numbers on the graph refer to the gene numbers in Additional file 2.

In order to confirm results obtained using the AppChip2 *A. pleuropneumoniae *microarray, eleven genes were selected for qRT-PCR analysis. Five of these were up-regulated *in vivo *(*sohB*, *hbpA*, *kpsF*, *apxIVA*, *phoR*) and six were down-regulated (*nlpI*, *visC*, *proQ*, *APL_1456*, *APL_1135 *and *nusG*). These genes represented a large range of log_2 _ratio values. In all cases, microarray results were supported by those obtained by qRT-PCR: all genes that were identified as up-regulated using the AppChip2 also showed up-regulation following qRT-PCR analysis, and the same was true for selected down-regulated genes (Figure 4).

#### b) Comparison with other in vivo studies

Among previous *in vivo *experiments that were conducted with *A. pleuropneumoniae*, the one that was closest to ours in terms of experimental conditions and results was by Baltes et al[18]. In their experiment, Selective Capture of Transcribed Sequences (SCOTS) was performed on lung tissues after a 7 day infection with *A. pleuropneumoniae *(end of acute phase), and 46 genes were identified [18]. Of the 150 differentially expressed genes that we have identified (see Additional file 1), 15 were also identified in other *in vivo *studies conducted with *A. pleuropneumoniae *(Table 2): only 4 of these were up-regulated following transcript profiling *in vivo*. These include 3 genes already identified by Baltes et al. using SCOTS at the end of the acute stage of the disease [18]: *fucI*, encoding the L-fucose isomerase, *nrdD*, encoding an anaerobic ribonucleoside triphosphate reductase, and *apxIVA*, encoding the ApxIVA toxin structural protein. The latter, the gene encoding the recently discovered fourth *A. pleuropneumoniae *Apx toxin [31], had only been detected *in vivo *[32-34] until it was discovered recently that it is also up-regulated after contact with broncho-alveolar lavage fluids [30]. It is also the only *apx *gene that was up-regulated in our experiment, but it is worth noting that the *apxIBCD *and *apxIIAB *genes, although not differentially expressed, were all actively transcribed *in vivo*. Finally, the last gene that was up-regulated in our study and identified in another *in vivo *study is *nanE*, which codes for a putative N-acetylmannosamine-6-phosphate 2-epimerase, an enzyme involved in the use of *N*-acetylneuraminate and *N*-acetylmannosamine as carbon sources [35]. The differences between our results and those obtained in other *in vivo *studies can be easily explained: while the second SCOTS experiment was conducted with animals suffering from chronic infections [10], a condition which differs greatly from our field case, the STM and IVET techniques do not provide a "snap-shot" of the transcriptome at a precise time point during the infection process. While STM and IVET can theoretically provide a global overview of transcriptomic events, the experiments conducted with *A. pleuropneumoniae *are not likely to have achieved this goal. Only 800 mutants were screened by Fuller et al. with IVET [12], well short of the approximately 2000 genes of the *A. pleuropneumoniae *genome, and although Sheehan et al. screened 2064 mutants using STM [16], the authors noted that both their STM screen and the previous STM experiment by Fuller et al. [15] were likely not saturating. STM relies on transposon mutagenesis, and there are some insertional hot spots in the *A. pleuropneumoniae *genome [16].

**Table 2:** Comparison between genes that were identified by transcript profiling *in vivo *(this study) and other *in vivo *techniques in *A. pleuropneumoniae*

Locus tag	Gene	TP	Other
APL_0030	*prfC*	↓	IVET (Fuller et al. 1999) STM (Sheehan et al. 2003)

APL_0771	*lpdA*	↓	STM (Fuller et al. 2000)

APL_1583	*cpxC*	↓	STM (Sheehan et al. 2003)

APL_1580	*cps5b*	↓	STM (Sheehan et al. 2003)

APL_0333	*visC*	↓	STM (Sheehan et al. 2003)

APL_1218	*Fur*	↓	STM (Sheehan et al. 2003)

APL_1388	*APL_1388*	↓	STM (Sheehan et al. 2003)

APL_0998	*apxIVA*	↑	SCOTS (Baltes et al. 2004)

APL_1684	*fuci*	↑	SCOTS (Baltes et al. 2004)

APL_0163	*nrdD*	↑	SCOTS (Baltes et al. 2004)

APL_1292	*APL_1292*	↓	SCOTS (Baltes et al. 2007)

APL_0282	*potC*	↓	SCOTS (Baltes et al. 2007)

APL_1474	*dnaG*	↓	SCOTS (Baltes et al. 2007)

APL_1401	*rpsL*	↓	SCOTS (Baltes et al. 2007)

APL_1752	*nanE*	↑	SCOTS (Baltes et al. 2007)

TP: Results obtained during transcript profiling

↑:Up-regulated

↓:Down-regulated

We also compared our results to the transcriptional profile of a virulent strain of *Pseudomonas aeruginosa*, another lung pathogen, isolated from a chronically infected cystic fibrosis patient [23]. There were no striking similarities when comparing the functions of differentially expressed genes in both studies. Since *P. aeruginosa *is well-known for the versatility of nutrients it can use for growth, and since both conditions (chronic infection vs acute infection) were highly different, this was to be expected. However, one interesting aspect of both cases was the list of genes that weren't up-regulated as predicted. As with Son et al., we noticed that few of the landmark virulence genes in *A. pleuropneumoniae *were up-regulated *in vivo*. Out of the three Apx toxins present in serotype 5b, only the gene encoding the ApxIV toxin was up-regulated. The Hsf autotransporter homolog, thought to be important in adhesion processes, as well as *pga *genes necessary for biofilm formation, were not identified either.

Well-characterized iron acquisition genes such as those coding for the hemoglobin receptor HgbA (despite the important hemorrhage noticed in the infected lungs) and the genes coding for transferrin binding proteins TbpA and TbpB did no show differential expression, nor did genes involved in anaerobic respiration. While in the latter case it can be argued that the environmental conditions encountered during the acute infection might explain why differential expression of these gene transcripts was not observed, it could also be hypothesized that years of evolution of the field strain in nature might have led to the loss of some regulatory mechanisms that could still be present in well-characterized laboratory strains, thereby leading to the constitutive expression of some important virulence genes. In the case of the *P. aeruginosa *clinical isolate, it was shown that more than 300 genes had higher levels of constitutive expression in the clinical isolate than in a well-characterised reference strain, therefore showing the deregulation of several pathways [23]. Whether this is also the case for *A. pleuropneumoniae *will have to be assessed.

#### c) Down-regulated genes

Interestingly, genes involved in protein synthesis were down-regulated *in vivo *towards the end of the acute phase of the disease. A total of 8 different 30S or 50S ribosomal genes showed levels of down-regulation ranging from -1.59 to -3.97 fold. These genes are scattered in the L20 genome, but all their respective operonic structures are well conserved when compared to those observed in *E. coli *[36]. Not much is known, however, about the regulation of these transcriptional units. While some experimental evidence seems to indicate that *rpsJ *could be activated by FNR [37] and repressed by ArcA [38], *rpsT *seems to be repressed by FNR [37]. Both *rpsK *and *rpsU *are transcribed from σ 70 promoters, which is the main sigma factor during the exponential growth phase [39]. This would probably indicate that *A. pleuropneumoniae *has a slower metabolism and replication rate *in vivo *than when it is growing in a rich culture medium. Further strengthening this hypothesis is the fact that two subunits from DNA polymerase III (*dnaN*, *dnaX*), which is the primary enzyme for replicative DNA functions in *E. coli*, are down-regulated, as well the genes coding for the DNA primase (*dnaG*) and the cell division protein FtsX.

With regards to their possible involvement in virulence processes, some genes that were down-regulated were of particular interest. The Fur transcriptional regulator, which represses transcription of genes associated with iron acquisition [40], showed a 2.6 fold down-regulation in transcription. In bacteria, Fur down-regulates the transcription of genes involved in iron-acquisition and iron homeostasis in bacterial cells, and its action is directly linked to the amount of available iron, as it requires Fe^2+ ^as a cofactor [41]. In *E. coli*, Fur also regulates its own level of expression. Iron restriction if often thought to be an important signal for bacteria, and has often been linked with the expression of virulence factors such as toxins [42]. Therefore, it would seem that the *in vivo *environment towards the end of the acute phase of pleuropneumonia is not iron-restricted for *A. pleuropneumoniae*. This is not surprising considering that there is extensive hemorrhage and tissue destruction, caused most probably by the secretion of Apx toxins. However, a few genes involved in iron acquisition, namely *APL_0096*, *APL_0668*, *hbpA *and *irp*, were up-regulated. Since these genes were not identified in a previous transcript profiling experiment under iron-restricted conditions [28], this might imply that they are not regulated by Fur. The *hfq *gene, which codes for a protein involved in RNA molecule stability and RNA-RNA interactions [43] and that therefore has an important regulatory function, was also down-regulated. Hfq is an RNA chaperone that acts as a post-transcriptional riboregulator [44]. The role of Hfq, a Sm-like protein, has been investigated with greater interest over the last decade since it was shown that it is involved in the function of small RNAs (sRNAs) [45]. It is believed to form a complex with sRNAs and RNAse E, thereby targeting mRNAs that are recognized by sRNAs for degradation. The fact that the *hfq *gene is down-regulated *in vivo *at the end of the acute phase of the disease could lead to a decline of the activity of sRNAs that require Hfq to function properly. No sRNAs have been identified in *A. pleuropneumoniae *yet, but they have been found in numerous bacterial pathogens [46].

Also of interest was the down-regulation of the *cps5b *(-2.21 fold) and *cpxC *(-1.82 fold) genes, involved respectively in the biosynthesis of capsular polysaccharide and in the export of these polysaccharides. Down-regulation of *cpxABC*, involved in capsule biosynthesis, has also been observed during planktonic life in liquid medium in contact with porcine lung epithelial cells [9]. The down-regulation of genes involved in capsule synthesis could potentially lead to the production of a thinner capsule on the surface of bacteria, thereby unmasking potential adhesins. Indeed, an acapsular mutant of *A. pleuropneumoniae *that was generated in our laboratory was shown to adhere more strongly to frozen porcine tracheal sections than the capsulated wild-type strain [47].

#### d) Up-regulated genes

Numerous genes that could be involved in the progression of the disease were identified as up-regulated. Gene *sohB *has the highest observed fold change (5.39×) and codes for a putative secreted serine protease that seems to be well conserved in other pathogenic bacteria, such as *Haemophilus parasuis*, *Neisseria meningitidis *and *N. gonorrhoeae*. In the lungs, *A. pleuropneumoniae *causes extensive tissue damage that could result from the combined action of Apx toxins and secreted proteases [48].

Adhesion processes are essential for the establishment of bacterial infections. The *apf *promoter was shown to be active *in vitro *during adhesion experiments to primary epithelial cells, and *in vivo *early in the infection process [49]. Here, we report that genes *apfB *and *apfC*, coding for the type IV pilin biogenesis proteins ApfB and ApfC, were up-regulated *in vivo *during natural infection by an *A. pleuropneumoniae *isolate. Type IV fimbriae, although primarily involved in promoting the attachment to biotic or abiotic surfaces, are also involved in many other processes, including DNA uptake, biofilm formation and twitching mobility [50]. Interestingly, another gene that could share similar functions was also up-regulated. *APL_0220 *codes for a member of the CscG superfamily of proteins involved in the assembly of curli fibers. Curli fibers are important for biofilm formation in numerous pathogens, including *E. coli *and *Salmonella enteritidis*, and are involved in the first steps of attachment. Curli-deficient strains tend to form flat biofilms [51]. CscG is the outer membrane protein that is responsible for the secretion of the curli structural subunits, and the *cscG *gene is generally found within an operon composed of the genes *cscDEFG*. In *A. pleuropneumoniae *L20, this organization is not respected: *APL_0220 *seems to form an operon with the genes *APL_0221 *and *APL_0222*. Neither of these two other genes shares homology with known *csc *genes. Finally, the *kpsF *gene, coding for an arabinose-5-phosphate isomerase, should also be mentioned since it is involved in the pathway responsible for the synthesis of 2-keto-3deozyoctulosonic acid (Kdo), which is present in the *A. pleuropneumoniae *LPS core oligosaccharide [52] and capsule of serotype 5 [53]. Studies in our laboratory have shown that it is the core oligosaccharide that is responsible for the previously observed LPS-associated adhesion mechanism [54,55].

Two operon structures that were up-regulated *in vivo *during the acute phase of the disease could give some insight into the environment the bacteria encounters in the host, and are the main reason why the "Transport and Binding Protein" functional class is so prominently represented in our list of up-regulated genes. Genes from the maltose operon, involved in the uptake and catabolism of maltose [56], were up-regulated: the genes *malP*, *malK*, *malF*, *malG*, *malQ *and a *malM *homolog (*APL_1234*) were actively transcribed in the host (average fold increase of 3.1), and gene *lamB1*, which codes for a maltoporin, showed a 5.14 fold change in the only hybridization in which the reporter wasn't manually flagged because of signal saturation. In *E. coli*, the *mal *genes are essential for the transport and utilization of maltose and maltodextrin [56]. Although it is not known whether maltose or maltodextrin are readily available as carbohydrate sources in mammalian lungs, it was previously shown that maltodextrin utilization is important for the colonization of the oro-pharynx in Group A *Streptococcus *[57,58]. It was also recently shown that catabolism of maltose provides a competitive advantage to pathogenic and commensal *E. coli *strains in the gut [59]. Meanwhile, genes from the *ula *operon (*ulaDCAG*), coding for a PTS transport system for ascorbate, showed an average fold change of 2.76. In *E. coli*, these genes are responsible for the transport and utilization of ascorbate in anaerobic conditions [60]. While ascorbate use has not been linked to bacterial virulence before, it has been shown on multiple occasions that genes involved in anaerobic metabolism are important for the virulence of *A. pleuropneumoniae *[10,61-63]. In pigs lungs, following extensive infection and tissue destruction, the bacteria are likely to face oxygen deprivation. A few other genes that are involved in anaerobic metabolism were up-regulated. The *hybB *gene codes for hydrogenase 2, a nickel-containing enzyme thought to be responsible for H_2_-dependent reduction of quinone under anaerobic conditions [64]. The phosphoenolpyruvate carboxylase and fumarate reductase subunit C, respectively encoded by genes *pepC *and *frdC*, are also required during anaerobic growth. However, other genes related to anaerobic metabolism and that were previously shown to be important *in vivo *[61-63], namely *dmsA*, *aspA *and *hlyX*, were not found in our list of differentially expressed genes. The *dmsA *gene did show a mean fold change of 1.60 over two successful hybridizations, but *hlyX *had a mean fold change of -1.66 over three successful hybridizations. However, neither of these changes were deemed statistically significant. This suggests that HlyX is probably not highly expressed during the acute phase of the disease caused by *A. pleuropneumoniae*. Accordingly, of 17 genes in our list that were previously shown to be up-regulated directly or indirectly by HlyX under anaerobiosis, 12 (*dnaX, visC, dsbC, mazG, amiB, cpxC, dacA, rpoZ, APL_0086, APL_1597, APL_1802 *and *APL_2043*) were down-regulated *in vivo*, while only 5 (*sohB, mglB, hybB, APL_0096 *and *APL_0920*) were up-regulated.

Two genes coding for proteins with regulatory functions were found to be up-regulated. The *phoR *gene codes for the phosphate regulon PhoR sensing protein, located in the inner membrane. In *E. coli*, this protein is part of a two-component regulatory system that responds to periplasmic orthophosphate concentration variations [65]. The phosphate regulon in *E. coli *controls the expression of at least 47 genes, and has been shown to be involved in virulence. Numerous *phoR*, *phoB *(the other component of the two-component system) and *pts *(the phosphate transport genes genetically and functionally linked to *phoBR*) mutants were found to be less virulent or avirulent when compared to wild-type strains, with phenotypes such as increased sensitivity to serum, acidity and cationic antimicrobial peptides [66] and reduced colonization [67]. *A. pleuropneumoniae *strain L20 harbours an operon composed of the genes *ptsSCAB*-*phoBR*. Although only *phoR *was deemed significantly up-regulated, all other genes showed levels of variations ranging from a 1.5- to 2-fold increase. Since it seems unlikely that porcine lungs are devoid of phosphate, the *A. pleuropneumoniae *PhoBR system might be important in order to adapt correctly to changing conditions inside the host. Another gene coding for a member of a two-component system was up-regulated: gene *APL_0628 *codes for a *cpxA *homolog. In different EPEC and UPEC strains, the CpxAR system was found to important for the correct folding and assembly of pili subunits [68-70]. However, the L20 *cpxA *homolog seems to either be truncated, or found on two separate gene loci. This seems to be the case since *cpxA *is found down-stream of *cpxR *(*APL_0629*), and gene *APL_0627 *is a hypothetical two-component sensor protein with a histidine-kinase domain.

#### e) Up-regulated genes which are conserved and predicted to code for OMPs or lipoproteins

The main goal of our experiment was to identify genes that were expressed *in vivo *during infection of the natural host. We were also interested by genes showing high-level of sequence conservation among different field and reference strains [25], and coding for proteins predicted to localize to the outer membrane [71]. Three genes satisfied those criteria: two that are coding for outer membrane proteins (*APL_0959 *and *irp*), and one coding for a lipoprotein (*APL_0920*) (see Additional file 1).

The *irp *(iron responsive protein) gene codes for a predicted TonB-dependant receptor, possibly involved in hemin transport. However, since this was deduced from sequence homologies, the real function of *irp *in *A. pleuropneumoniae *cannot be reliably inferred, but it is conserved in various bacterial pathogens. Interestingly, the *irp *gene is located directly next to *APL_0920*, a conserved lipoprotein in the L20 genome although they are transcribed in opposite directions. The protein sequence deduced from the *APL_0920 *gene sequence is present in all sequenced *A. pleuropneumoniae *strains and in *Mannheimia haemolytica *PHL213, but no other strong homologies can be found in other bacterial species.

The *APL_0959 *gene codes for a hypothetical hemagglutinin/hemolysin-like outer membrane protein. The protein sequence deduced from the gene sequence shares approximately 30% identity with different large sections of the filamentous hemagglitinin/adhesin (FhaB) from bacteria of the *Bordetella *genus, which also colonize the respiratory tract of various mammalian hosts. While it was first identified for its hemagglutination properties [72], FHA can bind carbohydrates, heparan sulphate and integrin [73]. It is the most important adhesin in *B. pertussis*, and deletion of FHA in *B. bronchiseptica*, another important swine respiratory pathogen, caused lower colonization than the wild-type strain at all respiratory tract sites tested with was unable to cause disease[74]. The *A. pleuropneumoniae *FHA homolog is conserved amongst all field and reference strains previously tested [25], and was predicted to locate to the outer membrane [71]. In *B. bronchiseptica*, FhaB is believed to be important early in the infection process in order to allow colonization of the ciliated epithelial cells of the upper respiratory tract. In *Histophilus somni*, the FhaB homolog is thought to also have an important role in biofilm formation in cases of myocarditis caused by this micro-organism [75]. Up-regulation of the *A. pleuropneumoniae fhaB *homolog during the acute phase of the disease hints at a similar role for this adhesin in the establishment of pleuropneumonia. Surprisingly, this gene was not identified as up-regulated in previous adhesion experiments performed in our lab [9], and it even showed a slight, although not significant, down-regulation when *A. pleuropneumoniae *adhered to immortalized lung epithelial cells. This can either reflect differences in regulatory events between a freshly isolated field strain and a well-conserved and characterized laboratory strain, or simply that *in vitro *experiments, however carefully designed they may be, can never really completely reproduce complex *in vivo *environments.

Despite years of research, an efficient cross-serotype vaccine that can prevent porcine pleuropneumonia has yet to be successfully produced. To generate a vaccine that provides good cross-serotype protection against porcine pleuropneumonia, one would most probably have to include antigens that are conserved amongst all serotypes that the host could encounter and be surface-exposed. The three proteins that are encoded by genes *fhaB*, *irp *and *APL_0920 *have all these features, and might therefore represent good candidates to include in future cross-serotype vaccines against *A. pleuropneumoniae*. FHA of *B. pertussis *is present in various combined diphteria-tetanus-pertussis (dTpa) acellular vaccines. These vaccines have been successfully used to prevent whopping cough and have contributed to a considerable decrease in the number of cases registered per year [76]. Thus the *A. pleuropneumonie *FhaB homolog represents an excellent candidate to include in a future pleuropneumonia vaccine that could actually lead to efficient protection against this important swine pathogen.

## Conclusions

To the best of our knowledge, this study represents the first and only genome-wide *in vivo *gene expression experiment to be conducted with *A. pleuropneumoniae *in its natural host, the pig, following a natural infection. Of the 150 genes that we could identify as differentially expressed, 72 showed greater levels of expression *in vivo *at the end of the acute phase of the disease than in a rich culture medium, while 78 were repressed. By comparing our results to those obtained following comparative genomics and proteomics experiments, we were able to identify three genes that were conserved in tested reference strains and in fresh field isolates of *A. pleuropneumoniae*, and these were also predicted to code for outer membrane proteins or lipoproteins. These three genes, namely *fhaB *(APL_0959), *irp *(APL_0919) and *APL_0920*, could all represent excellent candidates for the development of a protective cross-serotype subunit vaccine against *A. pleuropneumoniae*.

While our results can help decipher some of the adaptations *A. pleuropneumoniae *has to make once it infects the host, much more research is necessary in order to understand the whole picture of the disease. The acute stage and the chronic stage of the disease are probably highly different conditions, and it would be interesting in future works to compare our present data with the transcriptomic profile of *A. pleuropneumoniae *during a chronic infection.

## Methods

### Animals

Animals were cared for accordingly to the Canadian Council on Animal Care (CCAC) guidelines on the care and use of farm animals in research, testing and teaching [77]. Clinical cases of pleuropneumonia were diagnosed in finishing pigs of a conventional farrow to finish herd. Lung samples were recovered from commercial pigs (Landrace × Yorkshire × Duroc) weighting more than 90 kg that were showing severe clinical signs and were either euthanized on site by a veterinarian (one case) or died from the disease (two cases). Some lung samples were stored in PBS containing 10% v/v of a solution preventing RNA degradation (95% ethanol, 5% buffer-saturated phenol)[78] and frozen at -80°C for RNA extraction, while others were sent to the Diagnostic Laboratory of the Faculté de médecine vétérinaire (Université de Montréal) for bacterial culture and serotyping. Samples from all animals were paraffin-embedded, and 4 μm sections were HPS-stained for microscopic observations.

### Bacterial strains and growth conditions

The *A. pleuropneumoniae *field strain 896-07 was isolated directly from the infected lung tissues and was routinely grown in BHI medium (Difco) supplemented with either 15 μg/ml (agar) or 5 μg/ml (broth) of NAD. For the transcript profiling experiment, this strain was grown in BHI-NAD broth at 37°C in an orbital shaker until an optical density of 0.3 at 600 nm was reached in order to obtain our reference condition. For the comparative genomic hybridization experiments, *A. pleuropneumoniae *serotype 5b strain L20 gDNA was used as a reference.

### RNA extractions

Bacterial RNA from the *A. pleuropneumoniae *field strain was extracted directly from three samples of frozen necrotic porcine lung tissues from the pig that was euthanised on site using an acid-phenol-chloroform extraction protocol. Briefly, tissue samples of approximately 1 g were cut in small pieces for homogenization with 1.0 mm zirconia/silica beads with a Mini-BeadBeater (Biospec, OK). Supernatant was recovered and subjected to two centrifugation steps: a first one at 1000 × g for 5 min at 4°C to remove cellular debris, and another step at 5000 × g for 5 min in order to pellet bacteria and remaining debris. Pellets were resuspended in PBS and pooled, and 0.5 volume of a SDS lysis solution (2% SDS and 16 mM EDTA) pre-heated to 100°C was added. Samples were incubated at 100°C for 5 min. before being subjected to two hot-acid-phenol-chloroform (Ambion, Tx) extractions, followed by two chloroform/isoamyl alcohol (Fluka) extractions. RNA was precipitated in 0.6 volumes of isopropylic alcohol and 10% sodium acetate 3 M pH 5.2 overnight, and further treated with the MicrobENRICH kit and Turbo DNase (Ambion, Tx) as prescribed by the manufacturer to ensure that contaminating eukaryotic RNA and eukaryotic and bacterial DNA were eliminated from the samples. PCR reaction using primers specific for *A. pleuropneumoniae *gene *ompW *and for porcine mitochondrial DNA were carried out to ensure that no bacterial or host DNA was left in the sample. RNA quality was verified on gel and also with a Nanodrop spectrophotometer.

### Genomic DNA extraction

Genomic DNA was extracted from the field strain 896-07 and the L20 reference strain as previously described [25]. Briefly, bacteria were suspended in TE buffer and treated with lysozyme (2 mg/ml) and RNase A for 10 minutes at room temperature, followed by digestion with proteinase K (0.1 mg/ml) in the presence of 0.1% SDS (wt/vol) for 1 hour at 37°C, until complete lysis was achieved. Genomic DNA was then isolated by extracting twice with phenol-chloroform-isoamyl alcohol (25:24:1), and twice with chloroform, and was finally precipitated in ethanol.

### Microarray hybridization

For the comparative genomic hybridization (CGH) experiments, samples were labeled and hybridized to the AppChip2 microarrays as previously described [25]. Briefly, genomic DNA from the reference L20 strain and from the field strain 896-07 was nebulized to 0.4 - 1.2 kb by passing nitrogen gas through an AeroMist Nebulizer chamber (IPI Medical Products, Chicago, IL) at 15 psi for 1 min. A total of 5 ìg of fragmented DNA was fluorescently labeled using direct chemical coupling with the Label-IT (Mirus Corp., Madison, WI) cyanine dyes Cy3 (reference strain) and Cy5 (field strain) as recommended by the manufacturer. Labeling efficiency was assessed spectrophotometrically, and samples were then combined and hybridized overnight to the AppChip2. For a complete description of AppChip2, see Gouré et al. 2009 [25].

Transcript profiling experiments were conducted as previously described [28]. Briefly, we proceeded to indirectly label cDNA synthesized from 15 μg of RNA from our reference (field strain grown in BHI broth) and experimental conditions (bacterial mRNAs isolated from necrotic lung tissues of the euthanized pig) using a monofunctional NHS-ester Cy3 or Cy5 dye (Amersham). Labelling efficiency was assessed spectrophotometrically, and labelled samples were then combined and added to the *A. pleuropneumoniae *5b strain L20 microarrays for overnight hybridization. All microarray slides were scanned using a Perkin-Elmer ScanArray Express scanner. Our microarray data was submitted to the Gene Expression Omnibus [79] [GEO:GSE15911]. Experiments were conducted on three different days, using different *in vivo *and *in vitro *samples. In order to ensure that the dye bias effect was minimal, two self-self hybridization assays using amplified RNA (MessageAmp, Ambion, Tx, USA) extracted from lung samples were conducted.

### Bioinformatics

All bioinformatics analyses were performed with the TM4 Microarray Software Suite [80] as previously described [28]. Raw data was generated using Spotfinder v.3.1.1. The integrated intensities of each spot, equivalent to the sum of unsaturated pixels in a spot, were quantified and the integrated intensity of the local background was subtracted for each spot. The same operation was performed with the median spot intensities. Spots with bad morphology, high local background and signal saturation were manually flaged and excluded from the data set. Data was normalized with the MIDAS software tool using cross-channel Loess normalization. Spots with median intensities lower than 1000 were removed from the normalized data set. Intensities for duplicate spots were averaged to generate the final normalized data set [25,28].

For the CGH experiments, a threshold of ± 0.9 on a log_2 _scale was used to identify genes that are likely divergent in the field strain, or were deemed absent from the field strain if the log_2 _ratio values was less than -3 [25]. A total of two hybridizations were conducted. For the transcript profiling experiments. Brefly, the Significance Analysis of Microarray (SAM) algorithm [81], which is included in the MeV software, was used to generate a list of differentially expressed genes following three distinct hybridizations. Using a false discovery rate (FDR) of 4.25%, a list of 150 differentially expressed genes was generated; this value estimates the proportion of genes likely to have been identified by chance. Functional classification of these genes was conducted using TIGR's Comprehensive Microbial Resource (CMR) [82]. Proteins were assigned to their corresponding pathways using the MetaCyc Metabolic Pathway Database [36]. Homologies were assessed using Blast tools [83] hosted on the NCBI and TIGR servers. The strain dendrogram was generated using Dendroscope v2.2.2 built 9 [84], adding results from field strain 896-07 to data previously obtained [25].

### Real-Time quantitative RT-PCR

Microarray results for the transcript profiling experiments were verified by real-time quantitative RT-PCR (qRT-PCR), using the QuantiTect^® ^SYBR^® ^Green RT-PCR Kit (Qiagen) on the same RNA samples that were used for the transcript profiling experiments. Reactions were performed in triplicate with a 16-place Cepheid Smart Cycler^® ^System in a total volume of 25 μl. Oligonucleotide primers (see Additional file 2) were designed using PrimerBlast [85] and tested on genomic DNA extracted from the *A. pleuropneumoniae *896-07 field strain. To ensure that amplification with these primers resulted in single amplicon of the anticipated size, they were PCR tested before proceeding to qRT-PCR analysis. Primer pairs which amplified fragments of 195 to 205 bp with a melting temperature of 60°C were selected. Eleven genes (5 up-regulated, 6 down-regulated) were selected for analysis. Relative expression of each gene as determined by qRT-PCR was normalized to that of the *rluC *gene which showed a stable level of expression throughout the different microarray experiments (data not shown). Quantitative measures were obtained using the 2^-ΔΔCT ^method [86].

## Authors' contributions

VD designed the transcript profiling experiments, carried out downstream data analysis, and drafted the manuscript. MD participated in the study design, supplied the infected lung tissues and revised the manuscript. CG conducted the microscopy work and revised the manuscript. JHEN designed AppChip2 and helped with the downstream data analysis. JH participated in the study design and revised the manuscript. MJ participated in the conception and supervised the design of the study and revised the manuscript. All authors read and approved the final manuscript.

## Supplementary Material

Additional file 1 ***A. pleuropneumoniae*****genes which are differentially expressed in infected pig lungs (150 genes).** Individual genes and their corresponding locus tag are sorted according to their functional class and fold change. *q*-values as calculated by SAM are indicated in %. Click here for file

Additional file 2 **Oligonucleotide primers used for microarray result validation by qRT-PCR**. Click here for file
