# Supplementary material for: Transcriptional profiling of Actinobacillus pleuropneumoniae during the acute phase of a natural infection in pigs
Source: BMC Genomics. 2010 Feb 8;11:98. doi: 10.1186/1471-2164-11-98 (PMC2829017; doi:10.1186/1471-2164-11-98)
Supplement: Additional file 1 — A. pleuropneumoniaegenes which are differentially expressed in infected pig lungs (150 genes). Individual genes and their corresponding locus tag are sorted according to their functional class and fold change. q-values as calculated by SAM are indicated in %. [file 1471-2164-11-98-S1.DOC]

**Additional file 1: *A. pleuropneumoniae* genes which are differentially expressed in infected pig lungs (150 genes).** Individual genes and their corresponding locus tag are sorted according to their functional class and fold change. *q*-values as calculated by SAM are indicated in %.

| **Locus Tag** | | **Gene** | **Description** | **Fold** | ***q* (%)** |
| --- | --- | --- | --- | --- | --- |
| *Amino Acid Biosynthesis* | | | | |  |
|  | APL_2025 | *hisH* | Imidazole glycerol phosphate synthase subunit HisH | 4.230 | 1.31 |
|  | APL_0139 | *leuC* | 3-isopropylmalate dehydratase large subunit 2 | 3.077 | 0.00 |
|  | APL_2026 | *hisA* | Phosphoribosylformimino-5-aminoimidazole carboxamide ribotide isomerase | 2.976 | 0.00 |
|  | APL_1043 | *APL_1043* | Probable aminotransferase | 2.949 | 0.00 |
|  | APL_1862 | *aroQ* | 3-dehydroquinate dehydratase | -2.680 | 3.65 |
|  | APL_0319 | *metE* | Cobalamin-independant homocysteine transmethylase | -3.478 | 3.74 |
| *Biosynthesis of cofactors* | | | | |  |
|  | APL_0572 | *pdxS* | Pyridoxal biosynthesis lyase PdxS | 2.825 | 0.78 |
|  | APL_0776 | *ispE* | 4-diphosphocytidyl-2-C-methyl-D-erythritolkinase | 2.612 | 0.00 |
|  | APL_0822 | *ubiA* | 4-hydroxybenzoate octaprenyltransferase | 2.401 | 0.78 |
|  | APL_0535 | *thiG* | Thiazole biosynthesis protein ThiG | 2.280 | 0.78 |
|  | APL_0310 | *moeA* | Molybdopterin biosynthesis protein MoeA | 1.739 | 2.06 |
|  | APL_0903 | *folE* | GTP cyclohydrolase I | -1.626 | 3.65 |
|  | APL_1513 | *coaA* | Pantothenate kinase | -1.750 | 4.00 |
|  | APL_1008 | *hemX* | Putative uroporphyrin-III C-methyltransferase | -1.841 | 3.69 |
|  | APL_0333 | *visC* | Putative monooxygenase family protein | -2.823 | 0.00 |
| *Cell Envelope* | | | | |  |
|  | APL_1494 | *ftpA* | Fine tangled pili major subunit; DNA-binding ferritin-like protein (oxidative damage protectant) | 3.428 | 3.95 |
|  | APL_0387 | *kpsF* | Arabinose-5-phosphate isomerase; polysialic acid capsule expression protein | 2.548 | 0.78 |
|  | APL_1599 | *mrdA* | Penicillin-binding protein 2 | 2.478 | 3.30 |
|  | APL_0878 | *apfBC* | Fimbrial biogenesis protein | 2.205 | 3.94 |
|  | APL_1136 | *amiB* | Putative N-acetylmuramoyl-L-alanine amidase AmiB precursor | -1.616 | 4.06 |
|  | APL_1583 | *cpxC* | Capsule polysaccharide export inner-membrane protein | -1.819 | 4.06 |
|  | APL_1863 | *APL_1863* | Putative mannosyltransferase | -2.169 | 4.06 |
|  | APL_1580 | *cps5b* | Region 2 capsular polysaccharide biosynthesis protein | -2.208 | 3.79 |
|  | APL_1596 | *dacA* | D-alanyl-D-alanine carboxypeptidase fraction A; penicillin binding protein 5 precursor | -2.217 | 4.00 |
|  | APL_0873^+^ | *rlpB* | Putative rare lipoprotein B | -2.243 | 1.13 |
|  | APL_0681 | *APL_0681* | Putative soluble lytic murein transglycosylase precursor | -2.523 | 1.13 |
|  | APL_1597 | *APL_1597* | Possible rare lipoprotein A RlpA-like protein | -2.571 | 4.11 |
| *Cellular Processes* | | | | |  |
|  | APL_0766 | *rec2* | Recombination protein 2 | 2.909 | 3.30 |
|  | APL_0303 | *tolB* | Translocation protein TolB precursor | 2.883 | 0.00 |
|  | APL_0998 | *apxIVA* | RTX toxin protein ApxIV structural component | 2.121 | 1.22 |
|  | APL_0988 | *hktE* | Catalase | 1.735 | 3.70 |
|  | APL_1344 | *ftsX* | Cell division protein FtsX-like protein | -2.029 | 1.13 |
|  | APL_0118 | *cspC* | Cold shock-like protein CspC | -4.413 | 4.00 |
| *Central intermediary metabolism* | | | | |  |
|  | APL_1752 | *nanE* | Putative N-acetylmannosamine-6-phosphate 2-epimerase | 2.830 | 2.42 |
|  | APL_1755 | *nagB* | Glucosamine-6-phosphate deaminase | 1.708 | 0.78 |
|  | APL_1508 | *APL_1508* | Putative Rhodanese-related sulfurtransferase | -2.788 | 4.00 |
| *DNA metabolism* | | | | |  |
|  | APL_0370 | *recB* | Exodeoxyribonuclease V beta chain | 1.537 | 2.17 |
|  | APL_1474 | *dnaG* | DNA primase | -1.565 | 4.00 |
|  | APL_0265 | *dnaX* | DNA polymerase III subunit γ/τ | -1.595 | 3.74 |
|  | APL_1142 | *recX* | Regulatory protein RecX | -1.698 | 3.74 |
|  | APL_1170 | *priB* | Primosomal replication protein | -1.761 | 4.25 |
|  | APL_0002 | *dnaN* | DNA polymerase III subunit β | -1.763 | 4.25 |
| *Energy metabolism* | | | | |  |
|  | APL_1240 | *malQ* | 4-alpha-glucanotransferase | 3.293 | 0.78 |
|  | APL_1333 | *hybB* | Pative Ni/Fe-hydrogenase 2 b-type cytochrome subunit | 3.123 | 0.00 |
|  | APL_1698 | *ulaD* | Probable 3-keto-L-gulonate-6-phosphate decarboxylase | 3.054 | 3.94 |
|  | APL_0869 | *abgB* | Aminobenzoyl-glutamate utilization-like protein | 2.942 | 0.00 |
|  | APL_1232 | *malP* | Maltodextrin phosphorylase | 2.879 | 0.00 |
|  | APL_1701 | *ulaG* | L-ascorbate-6-phosphate lactonase UlaG-like protein | 2.452 | 1.31 |
|  | APL_1684 | *fucI* | L-fucose isomerase | 2.342 | 0.00 |
|  | APL_1019 | *kdgK* | 2-dehydro-3-deoxygluconokinase | 2.290 | 1.31 |
|  | APL_2011 | *aldA* | Putative aldehyde dehydrogenase AldA | 2.215 | 2.42 |
|  | APL_0452 | *sucC* | Succinyl-CoA synthetase beta chain | 2.209 | 2.35 |
|  | APL_0969 | *glnE* | Glutamate-ammonia-ligase adenylyltransferase | 2.003 | 2.14 |
|  | APL_0339 | *pepC* | Phosphoenolpyruvate carboxylase | 1.760 | 3.95 |
|  | APL_1527 | *frdC* | Fumarate reductase subunit C | 1.573 | 2.09 |
|  | APL_0375 | *glpK* | Glycerol kinase | 1.506 | 4.11 |
|  | APL_1450 | *fbp* | Fructose-1,6-bisphosphatase | -1.607 | 3.65 |
|  | APL_0771 | *lpdA* | Dihydrolipoyl dehydrogenase | -1.617 | 4.00 |
|  | APL_0607 | *nfnB* | Putative NAD(P)H nitroreductase | -1.661 | 3.69 |
|  | APL_1638 | *dsbA2* | Thiol disulfide oxidoreductase | -1.944 | 3.74 |
|  | APL_0755 | *fabI* | Enoyl-[acyl-carrier-protein] reductase (NADH) | -1.949 | 3.65 |
|  | APL_1652 | *atpB* | ATP synthase A chain | -1.959 | 4.00 |
|  | APL_1479 | *APL_1479* | Thioredoxin-like protein | -2.018 | 3.79 |
|  | APL_0644 | *p­ta* | Phosphate acetyltransferase | -2.995 | 4.13 |
| *Fatty acid and phospholipid metabolism* | | | | |  |
|  | APL_1689 | *APL_1689* | Probable alcohol dehydrogenase | 1.898 | 0.78 |
| *Mobile and extrachromosomal element functions* | | | | |  |
|  | APL_0524 | *APL_0524* | Predicted phage tail protein | -2.435 | 3.74 |
|  | APL_0984 | *APL_0984* | Putative transposase | -4.773 | 3.79 |
| *Protein fate* | | | | |  |
|  | APL_0008 | *sohB* | Putative secreted serine protease SohB | 5.390 | 2.02 |
|  | APL_1962 | *hflX* | GTP-binding protein hflX | -1.680 | 3.74 |
|  | APL_0458 | *dsbC* | Thiol:disulfide interchange protein DsbC precursor | -1.853 | 1.13 |
|  | APL_1509 | *secB* | Protein-export protein SecB | -2.115 | 4.30 |
|  | APL_1456 | *slyD* | FKBP-type peptidyl-prolyl cis-trans isomerase SlyD | -2.268 | 4.25 |
|  | APL_0743 | *secG* | Protein-export membrane protein | -4.155 | 3.79 |
| *Protein Synthesis* | | | | |  |
|  | APL_1759 | *rpsJ* | 30S ribosomal protein S10 | -1.592 | 3.74 |
|  | APL_0030 | *prfC* | Peptide chain release factor 3 | -1.734 | 3.74 |
|  | APL_0982 | *rpmE* | 50S ribosomal protein L31 | -2.172 | 2.49 |
|  | APL_1169 | *rplI* | 50S ribosomal protein L9 | -2.266 | 3.79 |
|  | APL_1473 | *rpsU* | 30S ribosomal protein S21 | -2.355 | 1.13 |
|  | APL_1401 | *rpsL* | 30S ribosomal protein S12 | -2.850 | 1.13 |
|  | APL_1972 | *rpmG* | 50S ribosomal protein L33 | -3.096 | 1.98 |
|  | APL_1558 | *rpsT* | 30S ribosomal protein S20 | -3.559 | 3.79 |
|  | APL_1782 | *rpsK* | 30S ribosomal protein S11 | -3.967 | 4.00 |
| *Purines, pyrimidines, nucleosides, and nucleotides* | | | | |  |
|  | APL_0775 | *prsA* | Ribose-phosphate pyrophosphokinase | 2.560 | 2.09 |
|  | APL_0163 | *nrdD* | Anaerobic ribonucleoside triphosphate reductase | 1.517 | 3.05 |
|  | APL_0646 | *cpdB* | 2',3'-cyclic-nucleotide 2'-phosphodiesterase precursor | -2.043 | 4.00 |
|  | APL_0682 | *hpt* | Hypoxanthine-guanine phosphoribosyltransferase | -2.293 | 3.79 |
|  | APL_0256 | *gmk* | Guanylate kinase | -2.569 | 4.13 |
| *Regulatory functions* | | | | |  |
|  | APL_0628 | *cpxA* | Putative sensor kinase CpxA | 1.997 | 4.30 |
|  | APL_0657 | *sspB* | Stringent starvation protein B; ClpXP protease specificity-enhancing factor | -1.598 | 3.97 |
|  | APL_0615 | *mlc* | NagC-like transcriptional regulator | -2.028 | 3.79 |
|  | APL_1961 | *hfq* | RNA-binding protein Hfq | -2.181 | 3.65 |
|  | APL_1218 | *fur* | Ferric uptake regulation protein | -2.612 | 4.25 |
| *Signal Transduction* | | | | |  |
|  | APL_1256 | *phoR* | Phosphate regulon sensor protein PhoR | 2.079 | 1.31 |
| *Transcription* | | | | |  |
|  | APL_1717 | *nusG* | Transcription antitermination protein NusG | -1.716 | 4.13 |
|  | APL_1826 | *rpoZ* | DNA-directed RNA polymerase omega subunit | -2.432 | 3.74 |
| *Transport and Binding Proteins* | | | | |  |
|  | APL_0855 | *hbpA* | Heme-binding lipoprotein A precursor | 3.649 | 0.00 |
|  | APL_0870 | *APL_0870* | Putative C4-dicarboxylate transporter | 3.414 | 0.00 |
|  | APL_1234 | *malM* | Maltose regulon periplasmic protein | 3.382 | 1.31 |
|  | APL_1700 | *ulaA* | Predicted ascorbate-specific permease IIC component | 3.155 | 4.11 |
|  | APL_1238 | *malF* | Maltose transport system permease protein MalF | 3.119 | 1.18 |
|  | APL_1239 | *malG* | Maltose transport system permease protein MalG | 3.073 | 2.17 |
|  | APL_1236 | *malK* | Maltose/maltodextrin import ATP-binding protein MalK | 2.936 | 0.00 |
|  | APL_1847 | *cysW* | Sulfate transport system permease protein cysW | 2.529 | 0.00 |
|  | APL_0848 | *APL_0848* | Putative ABC transporter periplasmic binding protein | 2.527 | 0.78 |
|  | APL_1699 | *ulaC* | Ascorbate-specific phosphotransferase enzyme IIA component | 2.404 | 3.95 |
|  | APL_0919* | *irp* | Iron-regulated outer membrane protein, TonB-dependant receptor protein | 2.255 | 3.13 |
|  | APL_0220 | *csgG* | Putative lipoprotein CsgG | 2.156 | 3.83 |
|  | APL_0369 | *norM* | Putative multidrug efflux protein, Na+/drug antiporter | 2.134 | 2.21 |
|  | APL_1665 | *gntP1* | Gluconate permease | 1.835 | 2.17 |
|  | APL_0096 | *APL_0096* | Zinc/iron transporter family protein ZIP | 1.815 | 3.05 |
|  | APL_0167 | *rnfC* | Electron transport complex protein RnfC | 1.699 | 1.31 |
|  | APL_0450 | *mglB* | D-galactose-binding periplasmic protein precursor | 1.659 | 4.11 |
|  | APL_1292 | *APL_1292* | Predicted ABC transporter ATP-binding protein | -1.549 | 3.69 |
|  | APL_0967 | *gltS* | Sodium/glutamate symport carrier protein | -1.672 | 3.65 |
|  | APL_1388 | *APL_1388* | Predicted ABC-transport permease | -1.829 | 3.65 |
|  | APL_1880 | *mscS* | Small-conductance mechanosensitive channel | -1.931 | 2.49 |
|  | APL_1457 | *aqpZ* | Aquaporin Z | -2.073 | 4.00 |
|  | APL_0282 | *potC* | Spermidine/putrescine transport system permease protein | -2.089 | 4.00 |
| *Hypothetical/Unknown/Unclassified* | | | | |  |
|  | APL_0920^+^ | *APL_0920* | Hypothetical protein | 4.469 | 0.00 |
|  | APL_0966 | *APL_0966* | Putative transport protein | 2.604 | 0.00 |
|  | APL_0668 | *APL_0668* | Predicted periplasmic lipoprotein involved in iron transport | 2.416 | 1.31 |
|  | APL_1188 | *APL_1188* | Hypothetical protein | 2.367 | 0.78 |
|  | APL_1934 | *APL_1934* | Hypothetical protein, conserved inner membrane protein | 2.302 | 0.00 |
|  | APL_0959* | *APL_0959* | Hemagglutinin/hemolysin-like protein; filamentous haemagglutinin outer membrane protein | 2.237 | 0.78 |
|  | APL_0999 | *APL_0999* | Hypothetical protein | 2.027 | 3.83 |
|  | APL_0141 | *APL_0141* | Hypothetical protein; possible H+/gluconate symporter | 1.965 | 3.83 |
|  | APL_1002 | *APL_1002* | Hypothetical protein | 1.920 | 0.00 |
|  | APL_1044 | *APL_1044* | Hypothetical protein; predicted permease rarD | 1.864 | 3.83 |
|  | APL_0815 | *APL_0815* | Hypothetical protein | 1.856 | 3.05 |
|  | APL_1082 | *arcD* | Putative arginine/ornithine antiporter | 1.818 | 4.09 |
|  | APL_0512 | *APL_0512* | Hypothetical protein | 1.777 | 0.78 |
|  | APL_0904 | *kdkA* | 3-deoxy-D-manno-octulosonic acid kinase | 1.745 | 3.01 |
|  | APL_1751 | *APL_1751* | Hypothetical protein; putative lipase or esterase | 1.548 | 3.83 |
|  | APL_0828 | *APL_0828* | Hypothetical protein, uncharacterized conserved protein | -1.631 | 3.97 |
|  | APL_1404^+^ | *oapB* | Opacity associated protein B | -1.771 | 1.13 |
|  | APL_2043 | *APL_2043* | Hypothetical protein | -1.882 | 3.74 |
|  | APL_0049* | *APL_0049* | Hypothetical protein | -2.001 | 4.00 |
|  | APL_0119 | *proQ* | proQ-like protein | -2.067 | 4.01 |
|  | APL_1135 | *APL_1135* | Hypothetical protein; putative ATPase | -2.164 | 3.74 |
|  | APL_1802 | *APL_1802* | Hypothetical protein | -2.181 | 3.97 |
|  | APL_0630 | *mazG* | Predicted pyrophosphatase | -2.235 | 3.79 |
|  | APL_1458 | *APL_1458* | Hypothetical protein; putative periplasmic/secreted protein | -2.249 | 4.00 |
|  | APL_1382 | *APL_1382* | Hypothetical protein | -2.374 | 3.94 |
|  | APL_0226 | *APL_0226* | Hypothetical protein; predicted kinase | -2.834 | 3.79 |
|  | APL_0428^+^ | *smpA* | Small protein A | -3.072 | 0.00 |
|  | APL_0359 | *nlpC* | Putative lipoprotein | -3.115 | 0.00 |
|  | APL_1639 | *slyX* | SlyX-like protein | -3.344 | 3.79 |
|  | APL_0653 | *csrA* | Carbon storage regulator CsrA | -3.444 | 3.65 |
|  | APL_0576 | *nlpI* | Lipoprotein NlpI-like precursor | -3.488 | 4.00 |
|  | APL_0086 | *APL_0086* | Hypothetical protein | -4.126 | 0.00 |

* Conserved outer membrane protein, as predicted by Gouré & al. and Chung & al.

^+^ Conserved lipoprotein, as predicted by Gouré & al. and Chung & al.
