# Supplementary material for: Transcriptional profiling of Actinobacillus pleuropneumoniae during the acute phase of a natural infection in pigs
Source: BMC Genomics. 2010 Feb 8;11:98. doi: 10.1186/1471-2164-11-98 (PMC2829017; doi:10.1186/1471-2164-11-98)
Supplement: Additional file 2 — Oligonucleotide primers used for microarray result validation by qRT-PCR. [file 1471-2164-11-98-S2.DOC]

**Additional file 2: Oligonucleotide primers used for microarray result validation by qRT-PCR**

| **#** | ***Gene*** | **Forward Primer** | **Reverse Primer** |
| --- | --- | --- | --- |
| 1 | *sohB* | ATGCGGTCATTTCTTTAGCG | ATTTTATTCGCTACGCACGC |
| 2 | *hbpA* | CACGCTATGCCAAACTGAAA | TCGGTAGCGTGTTGTAGTGC |
| 3 | *kpsF* | GTAAATTGCTCAATCGCGTG | AACTGTCCGTACCGAATTGC |
| 4 | *apxIVA* | CTGAATAAACCGGACGGAAA | CATGGTCGAATAACGCTCCT |
| 5 | *phoR* | GCATTTACCGCATTTAACCG | GTTTCACCAAGCAGCGATTT |
| 6 | *nlpI* | ATTAAATCCGGACGAACGTG | TAAAGGCATCAATCGCACTG |
| 7 | *visC* | GATTACGAGCAAACCGCATT | GCTCCCGATTAAATTGTTGC |
| 8 | *proQ* | AGAAGCCGGTATTGTGGATG | GAAGCTTCTTTCGCAACTCG |
| 9 | *APL_1456* | GGAAGAAGGTTACGGCGAAT | ACTCTTGCCCGGCTAACATA |
| 10 | *APL_1135* | TTAAACGGTGAACTTGGTGC | CGAATCCCCATAAACTCCAA |
| 11 | *nusG* | ACCGACCGAAGAAGTTGTTG | AAAATACGATCCGCTTCACG |
| Ref | *rluC* | TACAGAACAGTTGCGGAAA | CATTCGCATAACGCTCTTCA |
|  | *Pig mito.* | GGCCACATTAGCACTACTCAACATC | AGATCCGATGATTACGTGCAAC |
|  | *ompW* | ggcgaagtggcaaaagtaaa | caacacctaaattcgcaatcg |

# : refers to the annotation of the gene on Fig. 4
